# Characterization of the *Rosa roxburghii* Tratt transcriptome and analysis of *MYB* genes

**DOI:** 10.1371/journal.pone.0203014

**Published:** 2019-03-12

**Authors:** Xiaolong Huang, Huiqing Yan, Lisheng Zhai, Zhengting Yang, Yin Yi

**Affiliations:** 1 Key Laboratory of State Forestry Administration on Biodiversity Conservation in Karst Mountainous Areas of Southwestern China /Key Laboratory of Plant Physiology and Developmental Regulation/ School of Life Sciences, Guizhou Normal University, Guiyang, China; 2 School of Life Sciences, Guizhou Normal University, Guiyang, China; University of North Carolina at Greensboro, UNITED STATES

## Abstract

*Rosa roxburghii* Tratt (Rosaceae) has a fruit that is flavorful, economically valuable, and highly nutritious, providing numerous health benefits. Myeloblastosis (MYB) proteins play key roles in the development and fruit quality of *R*. *roxburghii*. However, there is little available genomic and transcriptomic information for *R*. *roxburghii*. Here, a normalized cDNA library was constructed from five tissues, including the stem, leaf, flower, young fruit, and mature fruit, using the Illumina HiSeq 3000 platform. *De novo* assembly was performed, and 470.66 million clean reads were obtained. In total, 63,727 unigenes, with an average GC content of 42.08%, were discovered, 60,406 of which were annotated. In addition, 9,354 unigenes were assigned to Gene Ontology categories, and 20,202 unigenes were assigned to 25 Eukaryotic Ortholog Groups. Additionally, 19,508 unigenes were classified into 140 pathways of the Kyoto Encyclopedia of Genes and Genomes database. Based on the transcriptome, 163 unigenes associated with MYBs were detected. Among these genes, 75 genes were significantly expressed in the various tissues, including 10 R1 MYB, 42 R2R3 MYB, one R1R2R3 MYB, three R4 MYB and 19 atypical MYB-like proteins. The expression levels of the 12 *MYB* genes randomly selected for quantitative real-time PCR analysis corroborated the RNA sequencing results. A total of 37,545 microsatellites were detected, with an average expressed sequence tag–simple sequence repeat frequency of 0.59 (37,545/63,727). This transcriptome data improves our understanding of the role of *MYB* in *R*. *roxburghii* and will be valuable for identifying genes of interest.

## Introduction

*Rosa roxburghii* Tratt (Rosaceae), commonly known as chestnut rose, is a fruit crop and deciduous horticultural shrub that is mainly distributed in southwestern China. It has several nutritional and functional components, including polysaccharides, flavonoids, triterpenes, and also exhibits superoxide dismutase activity [1, 2]. Some pharmacological researches indicate that it could be used for the inhibition of the metastasis and invasion of ovarian cancer cells and senescence-retardation [3, 4]. In addition, its fruits exhibit radio-protective, anti-tumor, antimutagenic, and genoprotective activities [5–7]. *Rosa roxburghii* is also a traditional Chinese medicinal plant that is further processed into fruit juice, preserves, and fruit wine [8].

Myeloblastosis (MYB) proteins have been reported to significantly influence plant development and fruit quality [9, 10]. Among the different transcription factor families, MYBs form the largest and most functionally diverse superfamily and are involved in regulating cell activities and plant development [11]. The N-terminus of a MYB domain is composed of adjacent tandem repeats [10]. The repeat encodes 50–53 amino acid residues and contains helices forming a helix-turn-helix domain that interact with the major grooves of specific DNA sequences [12]. MYB superfamily members are classified into several subfamilies based on the number of conserved domains (R), including R1 MYB (with one R), R2R3 MYB (with two Rs), R1R2R3 MYB (with three Rs), R4 MYB (with four Rs), and atypical MYB-like proteins [13]. Many MYB superfamily proteins and their functions have been determined in different species. Due to the conserved domains, the R2R3 MYB type is predominant in plants [14]. MYBs are involved in regulating plant growth, development, and stress resistance, including the anthocyanin biosynthetic pathway, trichome initiation and development, flavonoid or phenylpropanoid metabolism, secondary wall biosynthesis, sugar signaling and responses to abiotic or biotic stress [15, 16].

The genes of the *R*. *roxburghii MYB* superfamily have not been comprehensively characterized. Thus, for species that lack reference genomes, high-throughput Illumina sequencing can be used to generate new transcripts, determine gene expression levels, and produce an accurate transcriptome profile [17]. Assembled unigenes with different database annotations can be assayed to evaluate genetic characteristics and metabolic pathways. Recently, the fruits of *R*. *roxburghii* at three different developmental stages were analyzed using Illumina sequencing [18]. The author detected several MADS-box genes related to fruit development, as well as NAD genes. Several transcripts related to ascorbate biosynthesis were also identified. However, the sequencing depth was insufficient to represent the entire transcriptome. In another study, genomic survey sequencing for the genetic background of *R*. *roxburghii* was also performed using next-generation sequencing technology by HiSeq 2500 sequencing. The estimated genome size was 480.97 Mb based on the ratio of K-mer number to peak depth, and the findings mainly illustrated the genes related to the biosynthesis of ascorbic acid [19].

To elucidate the transcriptomic profiles of different tissues in *R*. *roxburghii*, the leaf, stem, flower, young fruit and mature fruit were subjected to RNA sequencing (RNA-Seq) analysis. The Illumina platform was used to construct a cDNA library using these five tissues to obtain transcriptome information. *MYBs*, which were significantly expressed in the various tissues, were identified. Expressed sequence tag-simple sequence repeats (EST–SSRs) were used to assess the genetic diversity of *R*. *roxburghii*. These results provide a valuable resource for functional gene analyses, particularly that of *MYB* genes.

## Materials and methods

### Biological materials

**Fig 1:**
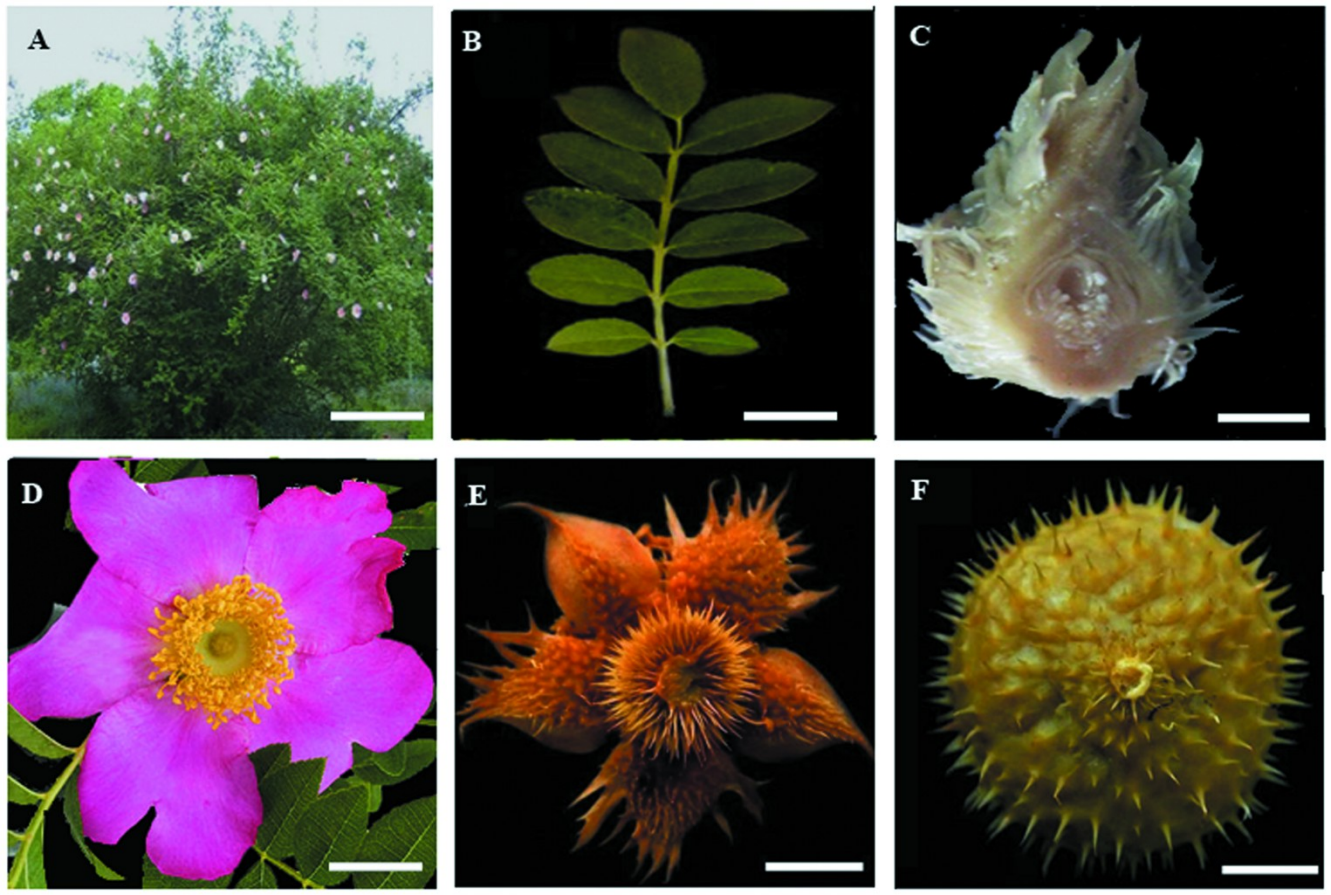
Photographs of the *Rosa roxburghii*. (A) plant, bar = 50 cm; (B) leaf and stem, bar = 0.5 cm; (C) flower bud, bar = 1mm; (D) flower, bar = 1 cm; (E) young fruit (50 days after flowering, DAF), bar = 1 cm; (F) mature fruit (120 DAF), bar = 1 cm.

Seedlings of wild *Rosa roxburghii* were provided by Hongying Zhou (Guizhou Botanical Garden, Guizhou, China) and planted outside in pots (one pot per seedling) under natural conditions at Guizhou Normal University, Guizhou province, China (N 26°42.408'; E 106°67.353'). Different tissues were collected from these plants exhibiting healthy growth, including the leaves, stems, flowers at the full-blossom stage, young fruit (50 days after flowering, YF) and mature fruit (120 days after flowering, MF). The former three samples were collected at the same time in May, 2017, while the latter two samples were respectively obtained in July and September (Fig 1A–1F). Three biological replicates were sampled for each tissue, resulting in a total of 15 samples. These materials were immediately frozen in liquid nitrogen following collection and then mechanically ground into a fine powder and stored at −80 °C until further analysis.

### RNA extraction and sequencing

A total of 0.1 g of frozen leaf, stem, flower, young fruit and mature fruit tissue were weighed. RNA was isolated using the Trizol method (Takara, Japan) with the addition of RNAiso-mate (Takara, Japan) according to the manufacturer’s guidelines. RNA quality was determined with a Nanodrop spectrophotometer (Wilmington, USA) and Agilent 2100 bioanalyzer (Santa Clara, USA). The total RNA was treated with RNase-free DNase I to eliminate DNA contamination. The enrichment of mRNA was performed with Oligo (dT)-attached magnetic beads, and fragmentation was performed using divalent cations under high temperature in NEBNext First Strand Synthesis Reaction Buffer (5×) to randomly interrupt mRNA. The first-strand cDNA chain was synthesized by random hexamers and M-MuLV Reverse Transcriptase (RNase H) with the mRNA template. Subsequently, the second-strand cDNA chain was synthesized by adding buffer, dNTPs, RNase H and DNA polymerase I. The the double-stranded cDNA was purified using AMPure XP beads. Following end-pair and single nucleotide A (adenine) addition for the purified cDNA, adapters were used to distinguish the different samples by selection with AMPure XP beads for PCR amplification, following which the cDNA library was obtained. Strand-specific cDNA libraries were constructed for sequencing using an Illumina HiSeq 3000 (Illumina, San Diego, USA) at Huazhong Agricultural University (Wuhan, China) to generate 150-bp paired-end reads.

### *De novo* assembly and functional annotation

The clean reads were trimmed by filtering out adaptor-only reads, reads with more than 5% unknown N bases, and low-quality reads (reads containing more than 50% bases with a Q-value≤10). *De novo* assembly was performed using the Trinity assembly program with default settings based on the de Bruijn graph algorithm [20]. Clean reads were first broken into shorter fragments (K-mers) and then assembled into longer fragments named contigs. The overlapping reads were mapped back to the corresponding contigs. Based on the paired-end reads, different contigs from the same transcripts were identified, and the distances among these contigs were calculated. These contigs were further assembled using Trinity to obtain sequences that were no longer defined as unigenes [20]. In addition, the assembled sequences that were less than 200 bp in size were removed. The assembled genome was used as the genomic reference. The raw sequence reads have been archived at the NCBI Gene Expression Omnibus (GEO) database with the accession number (GEO No. GSE122014).

To determine the function of the unigenes, BLASTx alignment with an E-value ≤ 10^−5^ was performed with different databases, including KOG (Eukaryotic Ortholog Groups, http://www.ncbi.nlm.nih.gov/KOG/) [21], Nr (NCBI non-redundant protein database, http://www.ncbi.nlm.nih.gov/), KEGG (Kyoto Encyclopedia of Genes and Genomes, http://www.genome.jp/kegg/) [22], Gene Ontology (GO, http://www.geneontology.org/) and Swiss-Prot (http://www.expasy.ch/sprot) [23, 24]. Based on the Nr annotations, the program Blast2GO [25] was applied to acquire the GO terms of the unigenes. After the GO annotations were obtained, WEGO software was used to perform GO functional classification and identify the distribution of gene functions in *R*. *roxburghii* at the macro level [26]. The best-aligned results were used to identify the sequence direction of the unigenes. If different databases conflicted, the results were prioritized in the following order: Nr, Swiss-Prot, KEGG, COG and GO [27]. When transcripts did not align to any of the databases, EST scans (http://myhits.isb-sib.ch/cgi-bin/estscan) was conducted to decide the sequence direction.

Homology with other model *Rosa* species was also assessed. Nucleic acid sequences from strawberry (*Fragaria X ananassa*) (*Fragaria ananassa* GDR RefTrans V1), apple (*Malus x domestica*) (*Malus x domestica* GDR RefTrans V1) and cherry (*Pruns*. *avium*) (*Prunus avium* GDR RefTrans V1) and *R*. *roxburghii* sequences were aligned using BLAST in the Uni-prot database. This was done in order to compare the four species based on exactly the same search parameters and database type.

### Identification and conserved motif analysis of *Rosa roxburghii* superfamily

Bowtie2 [28] and RSEM (RNA-Seq by Expectation-Maximization) [29] were used to map clean reads to the assembled transcriptome and evaluate the abundance of each transcript, respectively. To compare the expression levels of transcripts in the different samples, FPKM (fragments per kilobase of transcript per million mapped reads) was used to normalize the gene expression [30] according to the formula: FPKM = (10^6^ × *C* × 10^3^)/*NL*. *C* represents the number of reads uniquely aligned to a certain unigene, *N* indicates the total number of reads uniquely aligned to all unigenes, and *L* is the base number of this unigene.

All genes related to MYBs were selected and analyzed based on Nr annotation and then described. The expression level of all *MYB* in the various tissues were assessed. The MYBs were predicted using Open Reading Frame (ORF) in TransDecoder (v2.1.0) (http://transdecoder.github.io/) with default parameters to obtain the protein. The longest ORF was used to identify the transcription sequence. Conserved motifs shared by MYB proteins, which were significantly expressed using a threshold value of absolute log_2_ FC (fold change) ≥1 with an FDR (false discovery rate) ≤ 0.01, were analyzed using Multiple Em for Motif Elicitation (MEME Version 5.0.2, http://meme-suite.org/tools/meme) online tool by uploading the amino acid sequences of the MYB superfamily members. The following parameter settings were applied: R1-MYB proteins, with one R; R2R3-MYB proteins, with two Rs; R1R2R3-MYB proteins, with three Rs; R4-MYB proteins, with four Rs. Others belonging to atypical MYB families were determined [31].

### Quantitative real-time PCR (qRT-PCR) assays

Twelve cDNAs encoding MYB transcription factors, all of which have potential roles in the regulation plant development, were selected for qRT-PCR validation. Primers were designed (S1 Table) with primer premier 6. Total RNAs were isolated from leaf, stem, flower, young fruit (YF) and mature fruit (MF) using the TRIzol, followed by purification with an RNA purification kit with added RNAiso-mate. The RNA was then reverse-transcribed using an RT-PCR Kit (TaKaRa, Japan) with an oligo dT-adaptor primer according to the manufacturer’s protocol. The volume of each reaction was 20 μL prepared with 10 μL of SYBR Green Master mix (Toyobo, Osaka, Japan), 1 μL of each primer pair, and 1 μL of cDNA template, and 7 μL sterilized ddH_2_O. The qRT-PCR reactions were performed in a Roche LightCycler480 machine with *β-actin* as an endogenous control. Three biological replicates were tested. Amplification was performed for 95°C for 2 min, followed by 40 cycles at 95°C for 15 s, annealing at 58°C for 30 s, and 72°C for 30 s. The expression levels relative to the control were estimated by calculating ΔΔCt and were subsequently analyzed using 2^−ΔΔCt^ method.

### Microsatellite detection

The program MISA (http://pgrc.ipksgatersleben.de/misa/) [32] was used to detect microsatellite repeat motifs for each unigene in order to determine the distributions of microsatellites (also known as SSRs) and to develop new markers in the transcriptome of *R*. *roxburghii*. The number of core repeat motifs in mononucleotide, di-nucleotides, tri-nucleotide tetra-nucleotide, penta-nucleotide and hexa-nucleotides was counted.

## Results

### Illumina sequencing and sequence assembly

**Fig 2:**
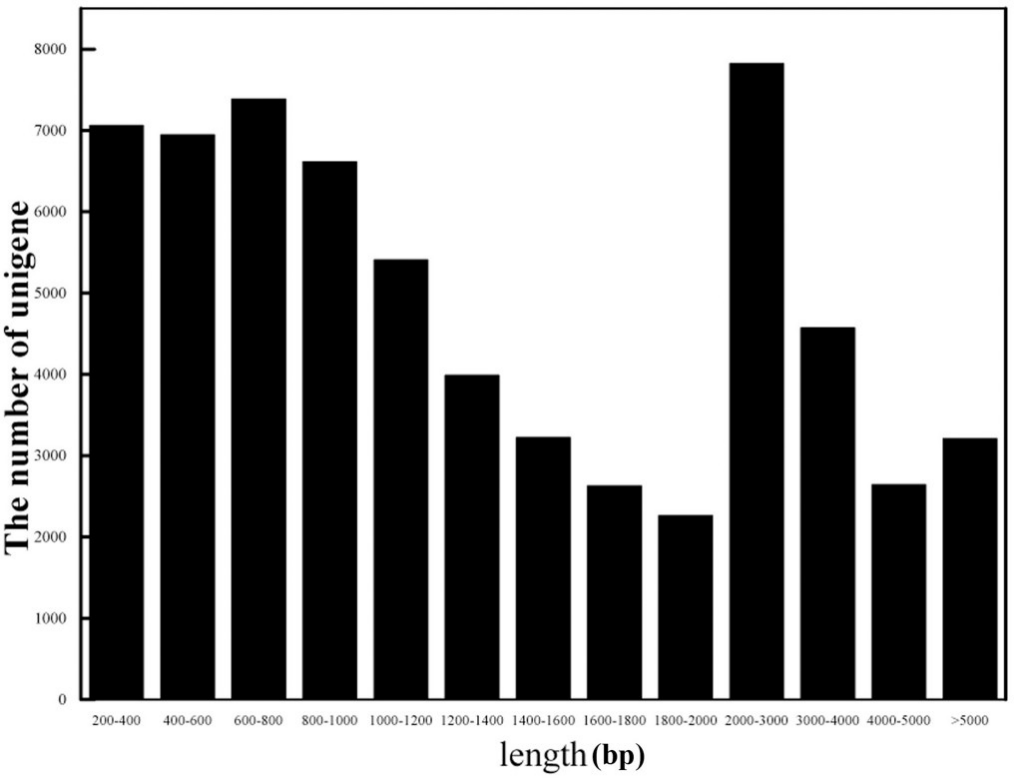
Length distribution of unigenes assembly for *Rosa roxburghii* Tratt.

A total of 470.66 million reads were obtained for all five tissues and all repetitions. After trimming and quality filtration of the raw data, each tissue was represented by an average of 31.38 million reads (S2 Table). The total reads per biological condition are indicated in S2 Table. For each sample, at least 86.21% of the reads could be mapped uniquely to contigs assembled using Trinity software. A total of 212,534 transcripts were obtained by assembling the clean reads using Trinity, with an average guanine plus cytosine (GC) content of 42.08%, an average length of 1437.69 bp and an N50 length of 2,085 bp. A total of 63,727 unigenes were assembled and all longer than 200 bp, with average and N50 lengths of 995 bp and 1,895 bp, respectively (Table 1). Of the 63,727 unigenes, 78.03% (49,727) were longer than 600 bp and 56.07% (35,732) were longer than 1 kb (Fig 2). In addition, most unigenes (60,901) were less than 5,200 bp (95.57%) (S3 Table and Table 1).

**Table 1:** Summary of the trinity assembly for *Rosa roxburghii*.

Length range (bp)	Transcript	Unigene
Total Number	212,534	63,727
Total length	305,558,508	85,677,327
N50 length	2,085	1,895
Median Length	1,077	995

### Functional annotation of unigenes

**Fig 3:**
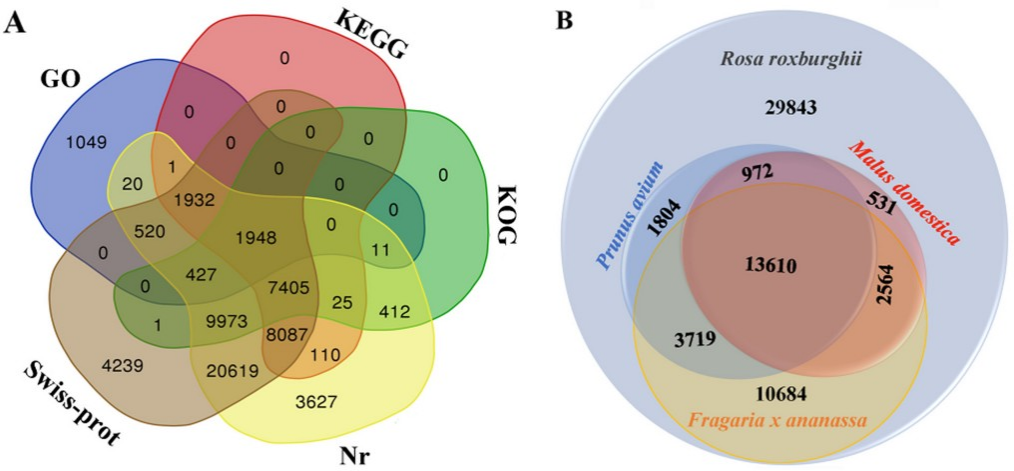
(A) Venn diagram of the number of unigenes annotated in five different databases. The number in the circles represents the number of unigenes annotated by single or multiple databases; (B) homology to strawberry (*Fragaria X ananassa*), apple (*Malus domestica*) and cherry (*Pruns avium*).

Using a BLASTX-based algorithm, 60,406 out of the 63,727 unigenes could be mapped to the GO, KOG, KEGG, Nr, and Swiss-Prot databases (S4 Table). Swiss-prot and Nr contained the highest number of homologous unigenes (55,151 and 55,118, respectively). In total, 1,948 unigenes could be annotated to all databases (Fig 3A). Comparative sequence alignment identified differences and similarities among the various *Rosa* species. Nucleic acid sequences from *Rosa* species, including strawberry, apple, and cherry, were aligned using a BLAST algorithm-based search of the Uniprot database. We found that strawberry had the highest number of similar genes, followed by cherry and then apple, with the numbers of unigenes matching those of *R*. *roxburghii* being 30,577 (47.98%), 20,105 (31.55%), and 17,677 (27.74%), respectively (Fig 3B).

**Fig 4:**
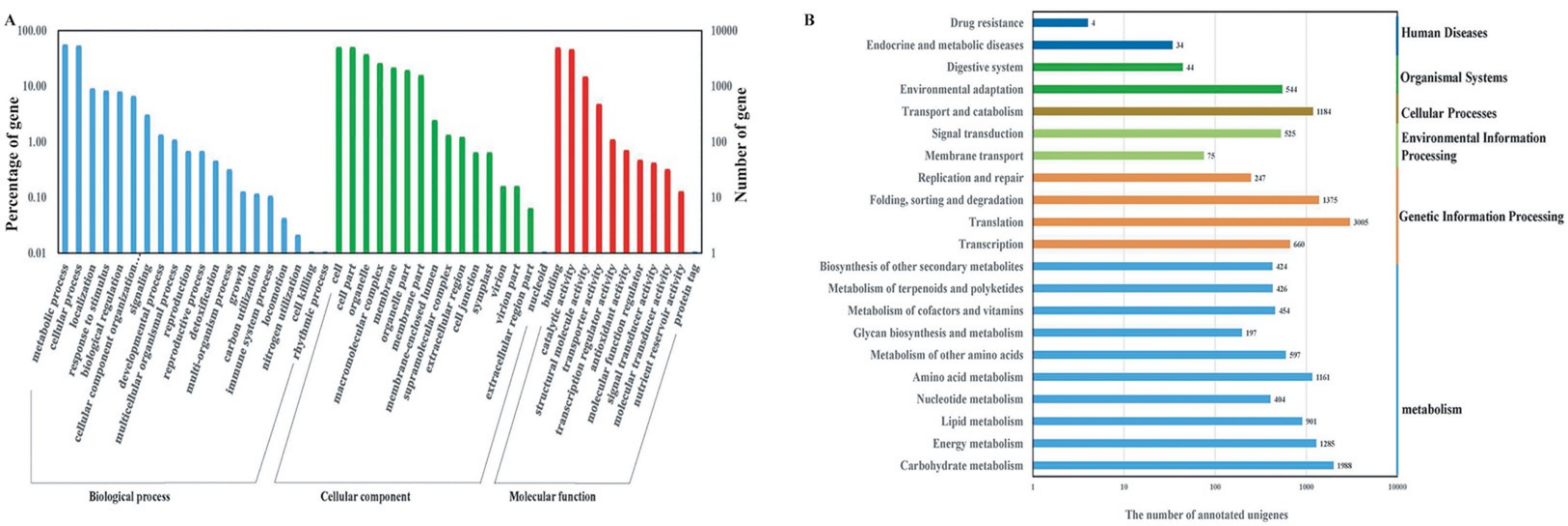
(A) GO functional classification of the unigenes. Blue indicates biological process, green indicates cellular process, and red represents molecular function. The axis labels are “Percentage of genes” (on the left) and “Number of genes” (on the right). (B) KEGG pathway analysis of the unigenes. The x-axis indicates the number of unigenes in a specific category. The left y-axis indicates the clustered functional groups, and the right y-axis indicates the specific category of the genes in the main category.

There are three GO categories: biological process, cellular component, and molecular function (S5 Table). The Category “biological process” consisted of 20 functional groups, with the major groups being metabolic process (56.56%) and cellular process (54.02%), followed by localization (9.10%) and response to stimulus (8.38%). For the category “cellular part”, 16 groups were predicted, with cell (49.89%), cell part (49.80%), and organelle (37.24%) constituting the three major groups. For “molecular function”, binding (49.02%) and catalytic activity (46.01%) were the dominant groups, followed by structural molecule activity (14.89%) (Fig 4).

A total of 20,202 unigenes were identified using the KOG database (S1 Fig) and annotated to 25 functional categories. General function prediction (46.37%) was the largest group, followed by signal transduction mechanisms (24.26%), posttranslational modification, protein turnover, and chaperones (23.49%), and translation, ribosomal structure, and biogenesis (19.80%). The numbers of unigenes assigned to transcription (11.81%), carbohydrate transport and metabolism (11.70%), energy production and conversion (11.67%), and intracellular trafficking, secretion, and vesicular transport (11.29%) were almost identical. In addition, lipid transport and metabolism represented 10.29%. However, there were still 1,939 unigenes with unknown functions. KOG classifications revealed the potential biological functions and provided an insight into the chemical reactions involved in the molecular processes in *R*. *roxburghii*.

A total of 19,508 annotated unigenes were assayed to determine the biological pathways represented in *R*. *roxburghii*. Briefly, these unigenes matched 140 KEGG pathways, as summarized in S6 Table. Translation in genetic information processing (3,005) was the dominant pathway, followed by carbohydrate metabolism (1,988), folding, sorting and degradation (1,375), energy metabolism (1,285), transport and catabolism (1,184), amino acid metabolism (1,161), and lipid metabolism (901) (Fig 4). The KEGG pathways can provide new insights into the biological properties of *R*. *roxburghii* and contribute to the prediction of the higher-level complexity of cellular processes and organismal behavior.

### Genes involved with MYB transcriptional factors in five different tissues

**Fig 5:**
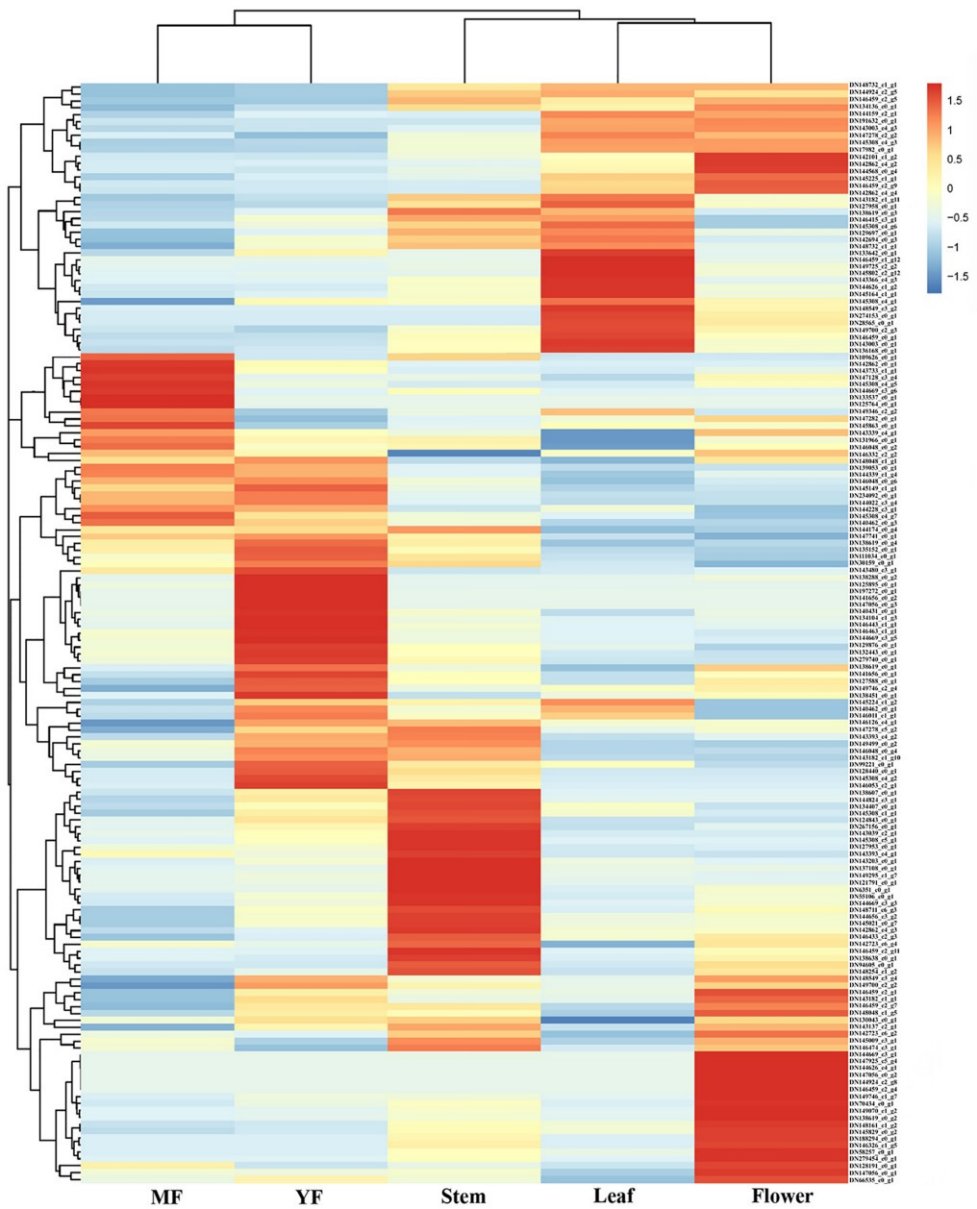
Heatmap and dendrogram indicating the expression levels of all *MYBs* using R software (version 3.1.3).

According to the functional database annotations, 163 *MYBs* were identified. Descriptions of the MYBs are listed in S7 Table, and 61 MYBs in *R*. *roxburghii* could be annotated with *Fragaria vesca*. MYBs regulate secondary metabolism and gene expression and are involved in environmental stress responses. The expression levels of 159 putative *MYB* genes in five tissues are indicated in a heatmap in Fig 5, produced in the R statistical environment (version 3.1.3), while four other genes (*DN141656 c0 g3*, *DN143182_c2_g1*, *DN145149_c1_g6* and *DN142101_c1_g3*) were not expressed in any tissues (S7 Table). Various *MYBs* exhibited differential expression among five tissues. Based on an expression value of log_2_ FC (fold change) ≥1 between any two tissues, there were 75 differentially expressed *MYBs* in the various tissues (S8 Table).

**Fig 6:**
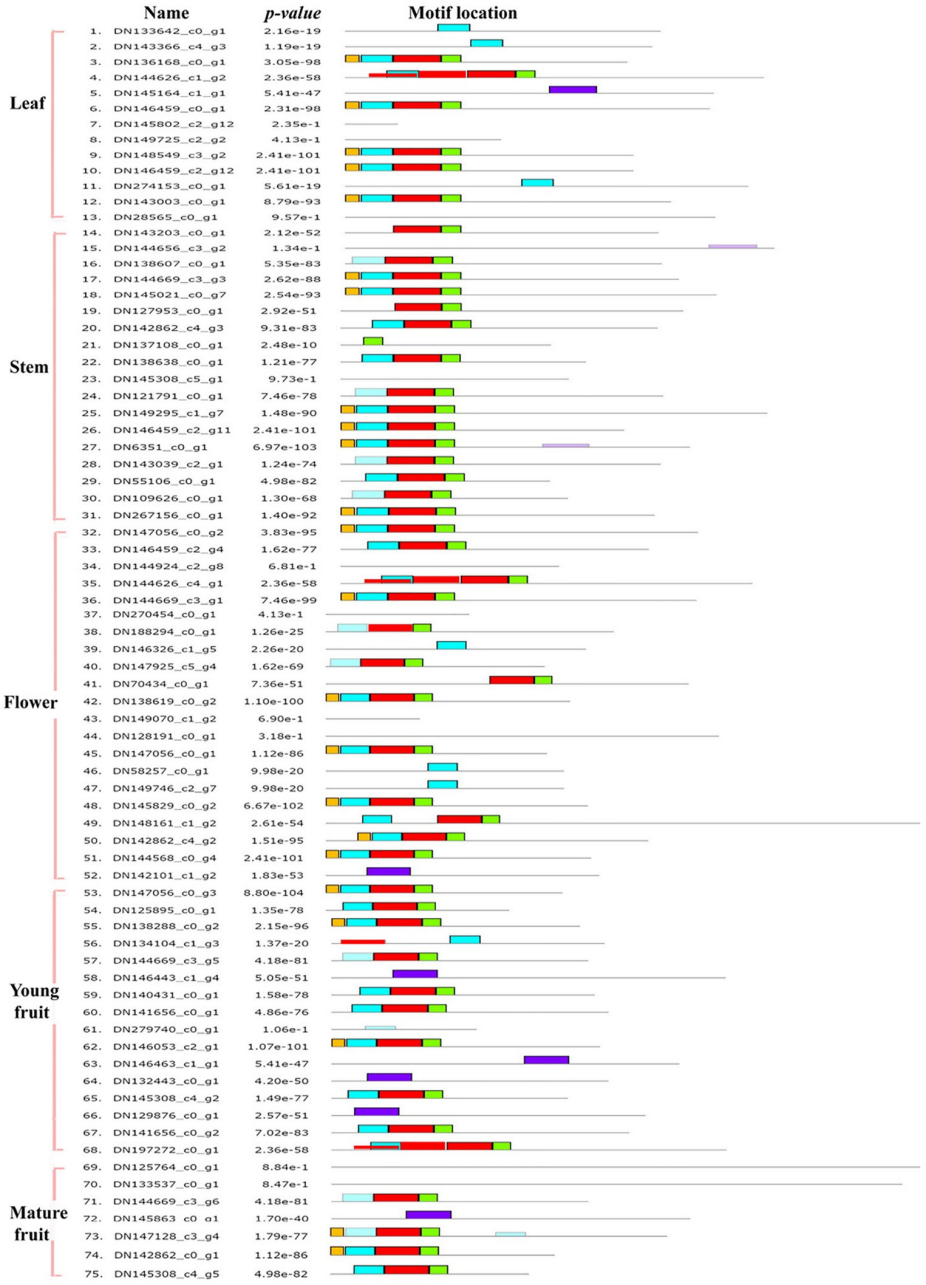
Seventy-five MYB motifs that were significantly expressed in the different tissues.

To investigate the features of homologous domains and each repeat MYB domain, the online MEME was used to search for the conserved motifs shared by these proteins by uploading the amino acid sequences. In total, 75 candidate MYBs were analyzed and are indicated in Fig 6. Ten R1 MYB, 42 R2R3 MYB, one R1R2R3 MYB, and three R4 MYBs were identified, while the remaining 19 proteins belonged to atypical MYB-like families (S8 Table). The sequences of the different conserved motifs are illustrated in S2 Fig

### Verification of RNA-seq results by qRT-PCR

**Fig 7:**
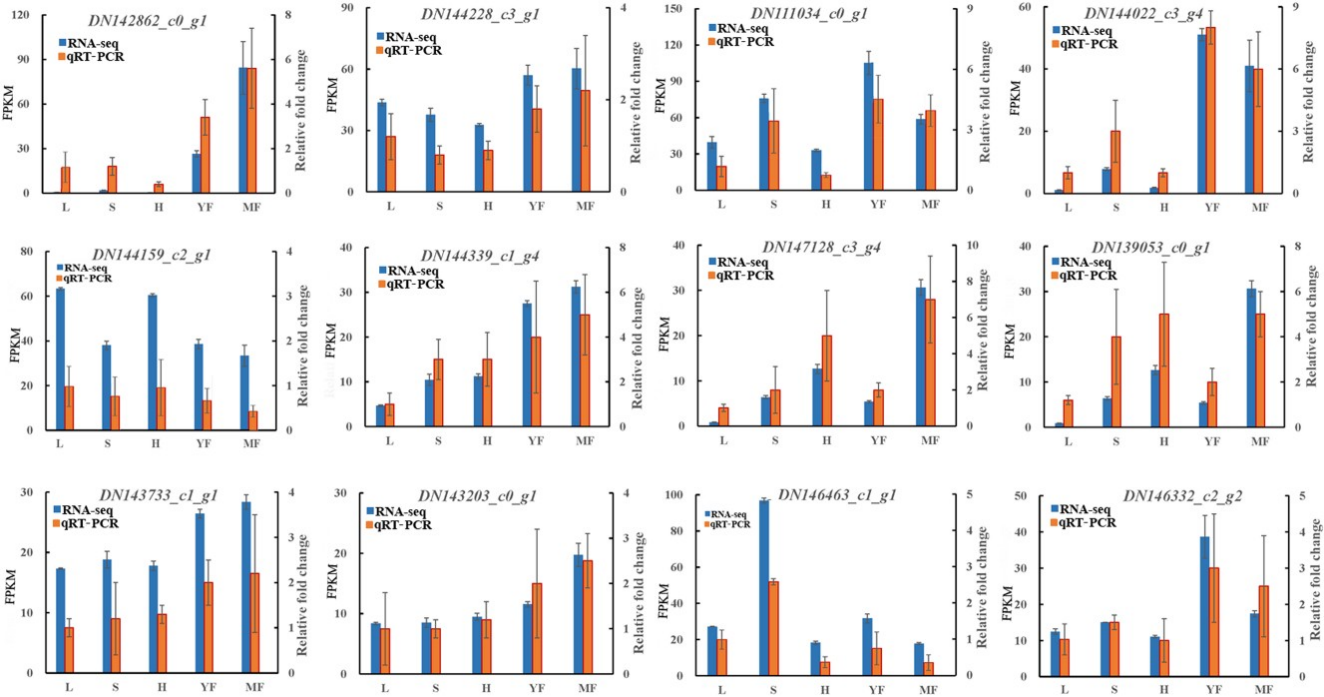
The relative expression levels of 12 randomly selected *MYB* by RNA-Seq and qRT-PCR. The horizontal axis indictes different tissues, including leaf (L), stem (S), flower (F), young fruit (YF) and mature fruit (MF). The left and right vertical axis individually indicate FPKM using RNA-seq and relative expression level using q RT-PCR. The error bars represent the standard deviation derived from each individual sample in triplicate.

qRT-PCR was conducted to validate the identification of the *MYB* genes obtained by RNA-seq analysis in the five different tissues. Using *β-actin* as the internal control, 12 genes related to MYB transcription factors were randomly selected. Validation results showed that the change trends of the 12 genes were nearly consistent with the gene expression patterns identified by RNA-seq (Fig 7), thereby confirming the reliability and accuracy of the RNA-seq analysis.

### Microsatellite analysis and SSR distribution

Using MISA software, 63,727 unigenes with a total length of 109,644,660 bp were screened for microsatellite determination. A total of 37,545 potential EST–SSRs were identified. The average distribution of the SSRs was calculated to be 1:2,920 bp (37,545/109,644,660), and the average frequency of an EST–SSR was 0.59 (37,545/63,727). In total, 20,321 unigenes contained one type of SSR (54.12%), and 8,757 contained more than one type of SSR (23.32%; S9 Table). In total, 5,275 (14.05%) SSRs were present in a compound formation. Transcriptome types of the SSRs, from single nucleotide to hexanucleotide, were abundant.

Among the identified 37,545 SSRs, repeats with mononucleotide motifs were the most abundant (19,589, 52.17%), followed by di-nucleotides (11.504, 30.64%) (Table 2). The most abundant motif was A or T (18,855, 50.22%), followed by AG or CT (8,055, 21.45%) (Table 2). Among SSRs with tri-, tetra-, and penta-nucleotides, the most abundant types were AAG/CTT (2,189, 5.83%), AAAT/ATTT (141, 0.38%), and AAAAT/ATTTT (19, 0.05%), respectively. The hexa-motifs AAGGAG/CCTTCT and ACCTCC/AGGTGG were the most abundant types and were equally present (7, 0.02%) (Table 2). The repeat positions of the SSR types were analyzed and ranged from 5 to 121. Most SSR types were repeated more than 15 times [19.01% (7,136)], while 17.10% (6,422) were repeated 10 times (Table 3). With the exception of mononucleotides, the repeat numbers for most SSRs ranged from 5 to 12 (9,612, 75.9%), with only a small percentage being repeated more than 15 times (1,177, 9.3%).

**Table 2:** Summary of EST-SSRs identified from the transcriptome of *Rosa roxburghii*.

SSR Type	Number	Percentage	Dominat motif	Number of dominant motif	Percentage of dominant motif in all EST-SSR
Mononucleotide	19,589	52.17%	A/T	18,855	50.22%
Dinonucleotide	11,504	30.64%	AG/CT	8,055	21.45%
Trinucleotide	5,879	15.66%	AAG/CTT	2,189	5.83%
Tetranucleotide	373	0.99%	AAAT/ATTT	141	0.38%
Pentanucleotide	93	0.25%	AAGAG/CTCTT	19	0.05%
Hexnucleotide	107	0.28%	AAGGAG/CCTTCT	7	0.02%
			ACCTCC/AGGTGG	7	0.02%

**Table 3:** Summary of different repeat times for SSRs isolated from the transcriptome of *Rosa roxburghii*.

SSR Type	5	6	7	8	9	10	11	12	13	14	15	>15	Total
Mononucleotide	0	0	0	0	0	5448	2864	2019	1380	1165	905	5808	19589
Dinonucleotide	0	2441	1765	1368	1158	883	985	858	216	259	263	1308	11504
Trinucleotide	2965	1385	727	461	91	86	69	40	20	8	7	20	5879
Tetranucleotide	242	95	15	7	4	5	0	3	2	0	0	0	373
Pentanucleotide	67	17	8	1	0	0	0	0	0	0	0	0	93
Hexnucleotide	74	22	7	1	1	0	1	1	0	0	0	0	107
Total	3348	3960	2522	1838	1254	6422	3919	2921	1618	1432	1175	7136	37545
Percentage	8.92%	10.55%	6.72%	4.90%	3.34%	17.10%	10.44%	7.78%	4.31%	3.81%	3.13%	19.01%	100%

## Discussion

The limited available genomic information for *R*. *roxburghii* has constrained previous genetic studies. The generation of RNA-Seq libraries using short-read Illumina sequencing technology is considered as an effective approach for assessing transcriptional expression in tissues of interest in species lacking reference genomes. In the present study, the clean reads of five tissues were used for library construction, and 470,657,040 clean reads were obtained with 150-bp paired-end reads. This is greater than in a previous transcriptome study of *R*. *roxburghii* [18]. Similar numbers of unigenes were assigned to GO categories as a previous study, while 19,508 unigenes were assigned to 140 KEGG pathways compared with the 7,480 unigenes assigned to 124 pathways in the previous study [18]. This is because more tissues were analyzed and more unigenes were annotated with a greater number of pathways in the present study. The GC content of *R*. *roxburghii* was calculated to be 42.05%, which is higher than the 38.63% detected in an earlier next-generation sequencing study [19]. A total of 63,727 unigenes were predicted with an average length of 1437.69 bp in our study, while 22,721 genes with an average length of 2311.52 bp were detected in the aforementioned study [19]. The greater volume of data in the present study increased the coverage depth and accuracy, allowing for the identification of a large number of genes involved in different metabolic pathways. Although five tissues with three repetitions each were used to construct cDNA libraries, this does not cover the entire transcriptome of *R*. *roxburghii*, as some transcripts, not expressed in these tissues, may be missed.

Based on the MYB expression patterns, 75 *MYBs* were significantly expressed in the various tissues. Among these *MYBs*, 42 R2R3 *MYB*s were identified, proving to be the dominant type. The MYBs presented similar patterns and conserved motifs, suggesting that their conserved features play similar roles in group-specific functions. For example, DN141656_c0_g3, which could constitute part of the MYB-bHLH-WD40 complex, is involved in plant trichome development [33]. Some R2R3 MYBs are related to secondary metabolism and responses to abiotic and biotic stresses [34–35]. Several R2R3 MYBs, such as DN111034_c0_g1 annotated as MYB12 and DN146459_c2_g7 annotated as MYB108, were analyzed for their possible involvement in flavonoid or phenylpropanoid biosynthesis and accumulation, as well as their regulation under different stress and hormone treatments [36–38]. MYBs are also reported to be implicated in sugar signaling, fruit-skin coloration, and anthocyanin metabolism [39–41]. The majority of the R2R3 MYBs detected in the present study are associated with plant growth and fruit development and could be further studied to improve fruit quality and stress resistance in *R*. *roxburghii*. Based on the similar functions of homologous gene with other *Rosa* species, the results of this study will aid functional studies of the *MYB* genes involved in *R*. *roxburghii* plant development.

A total of 37,545 microsatellites with different repeat types were detected from 63,727 unigenes, indicating that each unigene, on average, contained 0.59 SSR. The SSR locus density was 1:2,920 bp, compared with 1:4.00 kb in a previous study [18]. Various criteria and parameters for SSR detection, as well as the diversity of genomic structures and compositions can influence SSR density [42]. While a positive and significant association between genome size and SSRs motif length exists in gymnosperms, SSR density and length contribute less to genome size diversity in angiosperms [43], Errors in sequencing and assembly mistakes that resulted in mononucleotide SSRs were relatively low [44]. Except mononucleotides, the most common SSR motif was dinucleotide repeats in the transcriptome, which was similar to previously reported results. Here, AC/GT was the common type. In conclusion, detected EST–SSRs (37,545) are more closely associated with functional genes than genomic SSRs [45]. Therefore EST–SSRs could provide valuable information for genetic and genomic analyses [46].

In summary, a deep RNA-seq analysis was conducted on five tissues, and a total of 470.66 million reads were generated. In total, 63,727 unigenes were obtained using Trinity, of which nearly 94.79% (60,406) were successfully annotated. The results of this study have provided increased scope for the detection of genes involved in various metabolic pathways and have further elucidated the potential roles of *MYBs* in the different tissues in *R*. *roxburghii*. The detected microsatellites allow for the identification of genetic linkage mapping construction and maker-assisted selection.

## Supporting information

S1 Table Primers for real-time PCR. (XLSX)

S2 Table Summary of read mapping in leaf, flower, stem, young fruit and mature fruit with three repetitions. (XLSX)

S3 Table Analysis of size distribution of unigene for *Rosa roxburghii* Tratt. (XLSX)

S4 Table The number of unigenes annotated according to the NCBI non-redundant (Nr), Swiss-Prot, KOG, Gene Ontology (GO) and KEGG database in *Rosa roxburghii* Tratt. (XLSX)

S5 Table The number of unigenes annotated with GO and classified with three categories. (XLSX)

S6 Table The number of unigenes involved in KEGG pathways. (XLSX)

S7 Table Analysis of genes related to MYB transcription factor in leaf, stem, flower, young fruit and mature fruit of *Rosa roxburghii* Tratt. FPKM means fragments per kilobase of transcript per million mapped reads.
(XLSX)

S8 Table The tissues where *MYB* exhibited significantly differential expression based on a threshold value of absolute log_2_ FC (fold change) ≥ 1, and types of MYB superfamily were listed. (XLSX)

S9 Table Summary of different type EST-SSRs identified from the transcriptome. (XLSX)

S1 Fig Histogram of Eukaryotic Ortholog Groups (KOG) classification of assembled unigenes. (TIF)

S2 Fig Logo sequences of the conserved motifs obtained using the MEME by uploading the amino acid sequences of seventy-five MYB. The overall height of each individual stack represents the conservation of the sequence at that position. The Arabic numerals under the colored capital letters represent the position of each residue and the width of the motif. Each color of the English letters indicates a different type of amino acid residue.
(TIF)
