# Supplementary figures and images for: Characterization of the Rosa roxburghii Tratt transcriptome and analysis of MYB genes
Source: PLoS One. 2019 Mar 12;14(3):e0203014. doi: 10.1371/journal.pone.0203014 (PMC6414006; doi:10.1371/journal.pone.0203014)

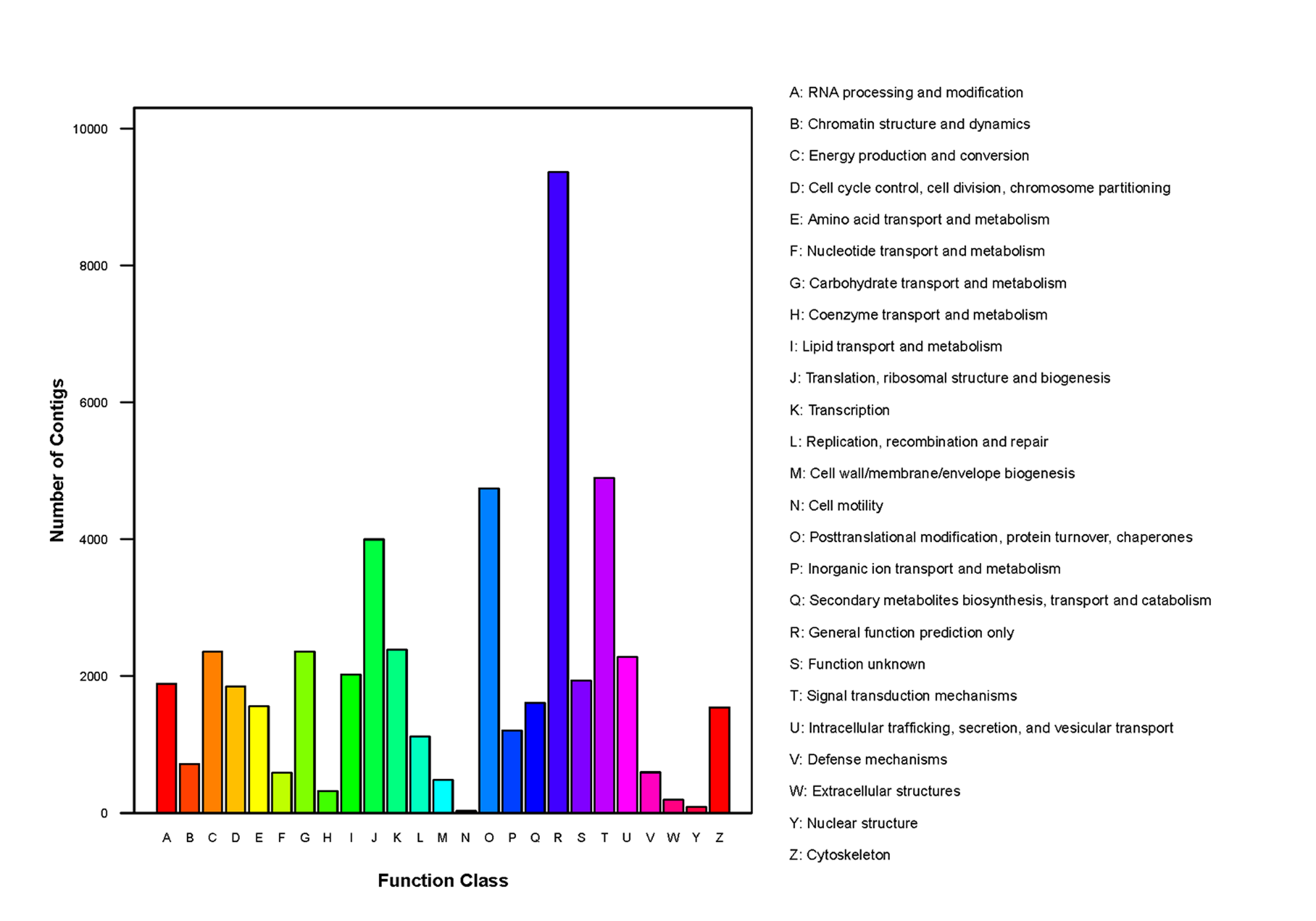

Supplement: S1 Fig — (TIF) [file pone.0203014.s010.tif]

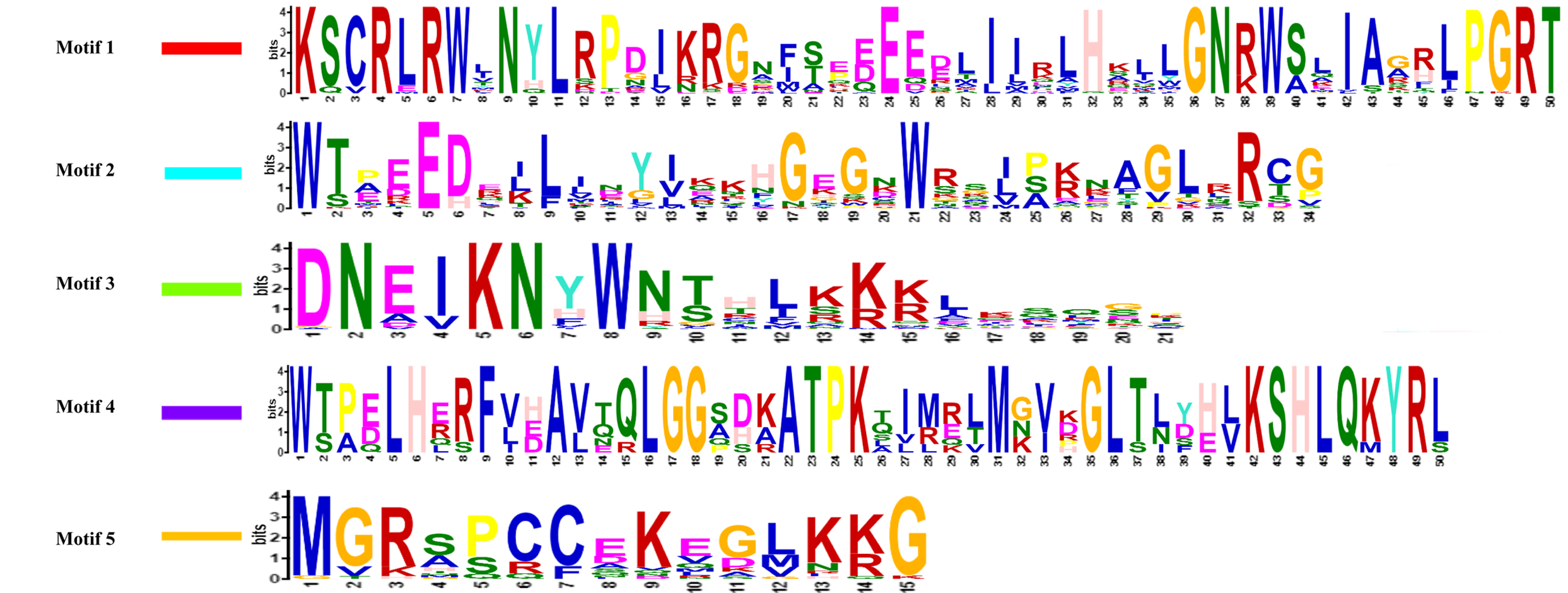

Supplement: S2 Fig — The overall height of each individual stack represents the conservation of the sequence at that position. The Arabic numerals under the colored capital letters represent the position of each residue and the width of the motif. Each color of the English letters indicates a different type of amino acid residue. (TIF) [file pone.0203014.s011.tif]
